# Definitions of Normal Liver Fat and the Association of Insulin Sensitivity with Acquired and Genetic NAFLD—A Systematic Review

**DOI:** 10.3390/ijms17050633

**Published:** 2016-04-27

**Authors:** Elina M. Petäjä, Hannele Yki-Järvinen

**Affiliations:** 1 Minerva Foundation Institute for Medical Research, 00290 Helsinki, Finland; hannele.yki-jarvinen@helsinki.fi; 2 Department of Medicine, University of Helsinki and Helsinki University Central Hospital, 00290 Helsinki, Finland

**Keywords:** insulin resistance, liver fat, obesity, PNPLA3, TM6SF2

## Abstract

Non-alcoholic fatty liver disease (NAFLD) covers a spectrum of disease ranging from simple steatosis (NAFL) to non-alcoholic steatohepatitis (NASH) and fibrosis. “Obese/Metabolic NAFLD” is closely associated with obesity and insulin resistance and therefore predisposes to type 2 diabetes and cardiovascular disease. NAFLD can also be caused by common genetic variants, the patatin-like phospholipase domain-containing 3 (PNPLA3) or the transmembrane 6 superfamily member 2 (TM6SF2). Since NAFL, irrespective of its cause, can progress to NASH and liver fibrosis, its definition is of interest. We reviewed the literature to identify data on definition of normal liver fat using liver histology and different imaging tools, and analyzed whether NAFLD caused by the gene variants is associated with insulin resistance. Histologically, normal liver fat content in liver biopsies is most commonly defined as macroscopic steatosis in less than 5% of hepatocytes. In the population-based Dallas Heart Study, the upper 95th percentile of liver fat measured by proton magnetic spectroscopy (^1^H-MRS) in healthy subjects was 5.6%, which corresponds to approximately 15% histological liver fat. When measured by magnetic resonance imaging (MRI)-based techniques such as the proton density fat fraction (PDFF), 5% macroscopic steatosis corresponds to a PDFF of 6% to 6.4%. In contrast to “Obese/metabolic NAFLD”, NAFLD caused by genetic variants is not associated with insulin resistance. This implies that NAFLD is heterogeneous and that “Obese/Metabolic NAFLD” but not NAFLD due to the PNPLA3 or TM6SF2 genetic variants predisposes to type 2 diabetes and cardiovascular disease.

## 1. Introduction

Non-alcoholic fatty liver disease (NAFLD) is defined as steatosis not caused by excess alcohol intake (>30 g/day in men and >20 g/day in women), hepatitis B or C, autoimmune hepatitis, iron overload, drugs or toxins [1]. It covers a spectrum from simple steatosis (NAFL) to non-alcoholic steatohepatitis (NASH) and cirrhosis [1,2]. NASH is characterized, in addition to steatosis, by ballooning necrosis, mild inflammation and possibly fibrosis, and can only be diagnosed using a liver biopsy [3].

Several longitudinal studies have shown that NAFLD increases the risk of and mortality from type 2 diabetes and cardiovascular disease [4]. Fibrosis stage is considered to be the most important histological feature predicting advanced liver disease [5,6]. It has been recently shown, however, that NAFL defined as macroscopic steatosis in more than 5% of hepatocytes progresses to NASH and fibrosis [7,8,9], as hypothesized by earlier indirect evidence [10]. Thus, NAFL predicts both metabolic and liver complications of NAFLD. It is therefore of interest to define normal liver fat content in humans.

Although NAFLD commonly coexists with obesity, insulin resistance and type 2 diabetes [11], common genetic causes also exist. A variant in patatin-like phospholipase domain-containing 3 (PNPLA3) (rs738409 [G], encoding I148M) confers susceptibility to NAFL, NASH and fibrosis (“PNPLA3 NAFLD”) [12]. Genetic variation in transmembrane 6 superfamily member 2 (TM6SF2) (rs58542926 [T], encoding E167K) is also increases liver fat and the risk of NASH (“TM6SF2 NAFLD”) [13]. These two conditions do not appear to be characterized by insulin resistance, although both genetic and metabolic causes of NAFLD may exist in the same person [14]. If so, then these types of NAFLD would not predispose to type 2 diabetes and cardiovascular disease.

The ensuing review will focus on defining normal liver fat content and discussing how liver fat content is related to insulin sensitivity in “Obese/Metabolic NAFLD” and the common genetic forms of NAFLD.

## 2. Definitions of Normal Liver Fat

### 2.1. Biochemical and Histologic Definitions

The biochemical standard for normal triglyceride content in the human liver is 5.5% of triglyceride of wet liver tissue weight [15,16]. Histologically, the liver is considered steatotic when ≥5% of hepatocytes in a tissue section stained with hematoxylin and eosin contain macrovesicular steatosis [17,18,19,20]. Steatosis is graded by the pathologist from 0 to 3 based on its severity: grade 0 (normal) = <5%, grade 1 (mild) = 5%–33%, grade 2 (moderate) = 34%–66%, and grade 3 (severe) = ≥67% of hepatocytes characterized by macroscopic steatosis [17]. As discussed below, these percentages seem quite different from those obtained by proton magnetic resonance spectroscopy (^1^H-MRS) (Table 1).

### 2.2. Proton Magnetic Resonance Spectroscopy (^1^H-MRS)

Steatosis can most accurately be measured using ^1^H-MRS [28]. This technique enables sampling of a large volume fraction of the liver compared to a biopsy [29,30] and provides an accurate and reproducible measurement of liver fat content [30]. However, ^1^H-MRS is expensive, as it requires use of magnetic resonance imaging (MRI) scanner and special expertise to perform proton magnetic resonance spectroscopy (^1^H-MRS) at the time of MRI scanning. ^1^H-MRS has been used in one population-based study, the Dallas Heart Study (DHS), to define normal liver fat content [23]. In this study, ^1^H-MRS was performed on 2349 subjects, of which 345 were considered healthy based on the following criteria: no history of liver disease or risk factors for hepatic steatosis (alcohol consumption ≤30 g/day in men, ≤20 g/day in women, body mass index (BMI) <25 kg/m^2^, normal fasting serum glucose, non-diabetic and normal serum alanine aminotransferase (ALT) (≤30 IU/L in men, ≤19 IU/L in women)). The upper limit of normal liver fat content was defined based on the upper 95th percentile in the healthy subjects and was 5.56% [23].

The ^1^H-MRS studies determine the hepatic triglyceride content rather than the percentage of hepatocytes with macroscopic lipid droplets. The relationship between ^1^H-MRS and histological liver fat content has been analyzed in two small studies, which included 13 [31], 12 [32] and 50 [33] subjects. In the first two studies, the ^1^H-MRS-determined normal liver fat in the DHS, *i.e.*, the 5.56% value corresponded to 15.7% [31] and 13.9% [32] of hepatocytes with macroscopic steatosis. On the third study, histological grade 1 (5%–33% macroscopic liver fat) corresponded to 11% (7%–14%), grade 2 (33%–66%) to 18% (14%–23%) and grade 3 (>66%) to 25% (10%–28%) ^1^H-MRS liver fat [33]. ^1^H-MRS-measured liver fat corresponds well to triglyceride content measured in a liver biopsy (*r* = 0.90, *p* < 0.001) [34]. These data show that the technique used to define normal liver fat influences the normal value.

### 2.3. Magnetic Resonance Imaging (MRI)

Hepatic steatosis can be diagnosed with MRI using an out-of-phase and in-phase imaging technique developed by Dixon WT *et al.* [35]. This method involves acquisition of MR images at echo times in which fat proton and water proton signals are either out-of-phase (water and fat signals cancel) or in-phase (water and fat signals add up) [35,36,37]. Once the out-of-phase and in-phase images are acquired by using constant calibration and other scanner settings, a quantitative fat signal fraction can be calculated from the hepatic signal [38]. Modified versions of the early Dixon method have been introduced. These include the hepatic fat fraction by Fishbein MH *et al.* which uses fast gradient echo techniques [25,39] and correlates well with histological liver fat content (*r* = 0.77, *p* < 0.001). The newer MRI-determined proton density fat fraction (PDFF) technique provides a quantitative, standardized and objective MRI measurement of hepatic fat based upon inherent tissue properties [40,41]. The MRI-PDFF method is reproducible and correlates closely with ^1^H-MRS (*r* = 0.99) [33,42] and liver histology (8.9%–9.4% at grade 1, 15.8%–16.3% at grade 2, and 22.1%–25.0% at grade 3, *p* < 0.0001) [33,43,44]. With this technique, the 5% macroscopic liver fat determined by histology corresponds to a PDFF value of 6% to 6.4% [45,46].

### 2.4. Ultrasound (US)

Ultrasound (US) is an inexpensive and widely available tool to visualize the liver and its fat content. Hepatic steatosis appears as a diffuse increase in parenchymal brightness and echogenicity on US images, and is often compared to hypoechogenity of the kidney cortex. Most studies score steatosis semiquantitatively as “mild”, “moderate” and “severe” based upon the visual assessment of hepatic echogenicity [27,47,48,49]. Lack of standardization precludes accurate comparison of data acquired by different machines and investigators. US lacks sensitivity in obese subjects [50] and in subjects with low liver fat content [51]. The sensitivity of diagnosing fatty liver increases from 55% to 80% when liver fat increases from 10%–20% to over 30% [51]. A recent study [52] suggested that the optimum sensitivity for US was achieved at a ^1^H-MRS-measured liver fat content greater than 12.5%. A meta-analysis of 44 studies comprising 4720 subjects concluded that US has a sensitivity of 85% and a specificity of 94% for detecting 20%–30% macroscopic steatosis [53]. The sensitivity and specificity were 65% and 81% for detecting 0%–5% steatosis and 93% and 88%, respectively, for detecting >10% steatosis.

Xia MF *et al.* created an equation for accurate quantification of liver fat content using US in Chinese subjects [54]. A tissue-mimicking phantom was used as a standard and the US hepatic/renal ratio was measured to calculate liver fat content in 127 subjects, in whom liver fat was also measured using ^1^H-MRS. The adjusted R^2^ for the model was 80%. The optimal cut-off for the US-measured liver fat content to diagnose hepatic steatosis was 9.15%, which yielded a sensitivity and specificity of 95% and 100%, respectively. The utility of this technique in other ethnic groups which are more obese than the Chinese in the face of a similar amount of liver fat [55,56] remains to be tested.

### 2.5. Computed Tomography (CT)

Hepatic steatosis can also be assessed by using computed tomography (CT) by comparing attenuation of the liver parenchyma to that of the spleen [57]. Tissue fat deposition lowers attenuation, hence fatty areas are less dense and appear darker than the non-fatty tissues [22]. The attenuation value in the healthy liver is 50 to 57 Houndsfield Units (HU) and 8 to 10 HU higher than that of spleen [22]. It decreases by 1.6 HU for every 1 mg of triglycerides per gram of liver tissue [58]. In subjects with steatosis, the mean attenuation value of the liver is lower than that of the spleen, and the liver appears darker than the spleen. Attenuation values less than 40 HU in the liver or 10 HU less in the liver than in the spleen are indicative of marked hepatic steatosis (>30%). Smaller fractions of fatty infiltration cannot be accurately and reliably assessed [59,60].

## 3. Non-Alcoholic Fatty Liver Disease (NAFLD) and Insulin Sensitivity

### 3.1. Insulin Resistance in “Obese/Metabolic NAFLD”

In subjects with NAFLD and the metabolic syndrome (MetS), *i.e.*, in “Obese/Metabolic NAFLD”, liver fat is closely correlated with direct measures of insulin resistance such as the inability of insulin to suppress hepatic glucose production [61], and indirect measures such as fasting serum insulin and the product of fasting insulin and glucose (Homeostasis model assessment for insulin resistance [HOMA-IR]) [62]. Indeed, liver fat correlates better with fasting insulin than with liver enzymes such as serum ALT and aspartate aminotransferase (AST) [63,64]. This close association between fasting insulin and liver fat is physiologically feasible as the main action of insulin after an overnight fast is to restrain hepatic glucose production. The inability of insulin to suppress hepatic glucose production increases fasting glucose, which stimulates insulin secretion leading to hyperglycemia and hyperinsulinemia.

**Figure 1:**
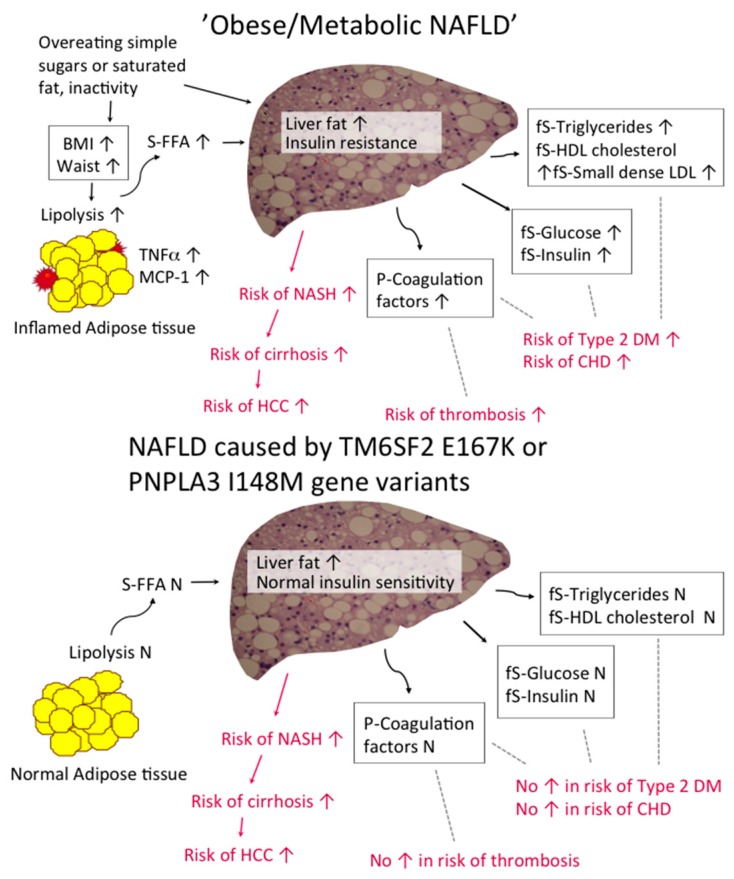
Schematic representation of causes and consequences of “Obese/Metabolic NAFLD” (**top**) and “TM6SF2 NAFLD” and “PNPLA3 NAFLD” (**bottom**). Abbreviations: BMI, body mass index; CHD, coronary heart disease; DM, diabetes mellitus; FFA, free fatty acids; fS, fasting serum; HCC, hepatocellular carcinoma; HDL, high density lipoprotein; MCP-1, monocyte chemoattractant protein-1; NAFLD, non-alcoholic fatty liver disease; NASH, non-alcoholic steatohepatits; LDL, low density lipoprotein; P, plasma; PNPLA3, patatin-like phospholipase domain-containing 3; S, serum; TM6SF2, transmembrane 6 superfamily member 2; TNF-α, tumor necrosis factor-α.

Lipolysis is the main source of fatty acids used for synthesis of intrahepatocellular triglycerides [65,66]. Liver fat is closely correlated with the ability of insulin to suppress lipolysis [67,68]. The ability of insulin to suppress very low density lipoprotein (VLDL) production is also impaired in NAFLD, which contributes to hypertriglyceridemia and a low high density lipoprotein (HDL) cholesterol concentration. Damaged hepatocytes release increased amounts of C-reactive protein (CRP) and coagulation factors, which could contribute to increased risk of cardiovascular disease and atherothrombotic vascular disease (Figure 1).

Any obese person with NAFLD and features of the MetS can be considered to have “Obese/Metabolic NAFLD” irrespective of genetic risk factors. The most recent proposal defines the MetS in 10 different ways [69]. The presence of any three out of five features (hypertriglyceridemia, low HDL cholesterol, hyperglycemia, hypertension, increased waist circumference) is required for diagnosis of the MetS [69]. For clinical practice, this definition still remains the best tool to diagnose insulin resistance, although the extent to which the 10 different definitions increase the risk of endpoints such as type 2 diabetes and cardiovascular disease is unclear. Measurement of fasting insulin and glucose concentrations and their calculation of their product HOMA-IR might seem more attractive direct tools to measure insulin sensitivity in subjects with NAFLD. The problem with this approach is that insulin assays are not internationally standardized and give highly variable results [70].

### 3.2. “Patatin-Like Phospholipase Domain-Containing 3 (PNPLA3) NAFLD” and Insulin Sensitivity

Approximately 30% of Europids and several other ethnic groups carry the PNPLA3 I148M variant [12]. The association between the PNPLA3 gene variant and NAFLD [12] has been replicated in over 50 studies, including eight genome wide association studies [71,72,73]. In a meta-analysis carriers of the I148M variant had 73% more liver fat, a 3.2-fold higher risk of necro-inflammation and a 3.2-fold greater risk of developing fibrosis than the non-carriers [71]. In a meta-analysis comprising 12 Asian studies, the risk of NAFLD was 1.9-fold increased in carriers compared to non-carriers [72]. Recent meta-analyses have also shown that this gene variant increases the risk of cirrhosis by 1.9-fold [74] and hepatocellular carcinoma (HCC) by 1.8-fold [75].

*In vitro*, the PNPLA3 I148M gene variant abolishes intrahepatocellular lipolysis [76,77] and by acting as a lysophosphatidic acid acyl transferase stimulates triglyceride synthesis from long unsaturated fatty acids containing coenzyme A (CoA) more than from saturated fatty acid CoAs [78]. The contribution of each these mechanisms to function of the PNPLA3 gene variant in the human liver is uncertain. It is clear, however, that the human liver lipidome markedly differs between “Obese/Metabolic NAFLD” and “PNPLA3 NAFLD” [14]. The increase in liver fat in the carriers of the PNPLA3 I148M gene variant is due to polyunsaturated triglycerides, whereas in “Obese/Metabolic NAFLD” the concentration of saturated triglycerides and insulin resistance-inducing ceramides is increased [14].

Table 2 summarizes the 14 studies that include data on insulin sensitivity in carriers and non-carriers of the I148M variant [12,79,80,81,82,83,84,85,86,87,88,89,90,91]. Carriers of the PNPLA3 I148M variant had more liver fat in their liver than non-carriers. Insulin sensitivity as evaluated by HOMA-IR [62], the hyperinsulinemic clamp technique, fasting or post-glucose insulin and glucose concentrations did not, however, differ between carriers and non-carriers of the gene variant. These studies included obese and non-obese, diabetic and non-diabetic as well as pediatric cohorts. Serum triglycerides were either similar or lower in variant allele carriers as compared to non-carriers, consistent with lack of insulin resistance (Table 2).

### 3.3. “Transmembrane 6 Superfamily Member 2 (TM6SF2) NAFLD” and Insulin Sensitivity

Approximately 7% of all subjects carry the TM6SF2 E167K variant. This gene variant increases the risk of NAFLD, independent of genetic variation in PNPLA3 at rs738409, obesity and alcohol intake [92]. A recent meta-analysis reported that carriers of the TM6SF2 E167K gene variant have a 2.1-fold higher risk of NAFLD than non-carriers [93]. They also had lower circulating total and low density lipoprotein (LDL) cholesterol, and triglyceride concentrations than non-carriers [93].

Four *in vitro* studies have examined the mechanism by which the TM6SF2 E167K gene variant could increase liver fat. Recombinant adeno-associated viral vectors expressing short hairpin RNAs were used to reduce *Tm6sf2* transcripts in the mouse liver, which increased hepatic triglyceride content three-fold [92]. TM6SF2 knock-out mice developed hepatic steatosis and had a three-fold reduced plasma VLDL triglyceride levels due to decreased lipidation [94]. In another study, TM6SF2 small interfering RNA inhibition also decreased export of triglyceride-rich lipoproteins and lipid droplet content in human hepatoma cell lines (Huh7 and HepG2) [95]. Overexpression of TM6SF2 in Huh7 cells reduced cellular triglyceride content [96]. Transient overexpression of human TM6SF2 in mice using a liver-targeting adenovirus containing the human TM6SF2 coding region increased, while knockdown of endogenous TM6SF2 decreased circulating total cholesterol [96]. In the latter study, no change in hepatic fat content was observed. This was hypothetized to be due to the transient exposure, compared to the lifetime exposure of humans carrying the gene variant [96].

Table 3 summarizes seven studies that have reported data on liver fat content and insulin sensitivity in carriers and non-carriers of TM6SF2 E167K gene variant [13,81,92,97,98,99,100]. In all but one of these studies, carriers had a significantly higher liver fat content as determined by ^1^H-MRS, MRI, histology or US [13,92,97,98,99,100] than non-carriers. Insulin sensitivity, as determined by HOMA-IR or from oral glucose tolerance test measures did not differ between carriers and non-carriers. Triglyceride concentrations were either lower [81,98,100] or similar [13,97,99] but also in one study higher [92] in TM6SF2 E167K variant allele carriers compared to non-carriers.

## 4. Materials and Methods

We performed a systematic search using PubMed and Medline on two topics. For definitions of normal liver fat, we used the following search terms and their combinations: “normal liver fat”, “liver histology”, “liver biopsy” and “liver triglycerides”, “liver H-MRS”, “liver MRI”, “liver MRI-PDFF”, “liver CT”, “liver ultrasound” and received 526 matches. Thirty-three studies included data on normal liver fat content or compared liver fat measured using different techniques. To review the association between insulin resistance and genetic NAFLD, we searched for studies using the following search terms: “PNPLA3” or “TM6SF2” and “insulin resistance”, “euglycemic (hyperinsulinemic) clamp”, “fasting glucose”, “fasting insulin”, “HOMA-IR”, “oral glucose tolerance test” and included studies which compared results between carriers and non-carriers of PNPLA*3* I148M or TM6SF2 E167K gene variants. A total of 124 matched were found. Of these, 22 studies were informative with respect to liver fat content and insulin resistance between genotypes, and were thus included.

## 5. Conclusions

Normal liver fat content based on liver histology can be defined as macroscopic steatosis in less than 5% of hepatocytes. With ^1^H-MRS, normal liver fat in the population-based DHS was defined as less or equal than 5.56% [23], which corresponds to histologic liver fat of approximately 15% [31,32]. Definitions of normal liver fat content thus depend on the method used. There is also no prospective evidence that these normal values are of clinical relevance with respect to the development of liver fibrosis.

Although NAFLD has often been regarded simply as the hepatic manifestation of the MetS, it is now clear that NAFLD is heterogeneous. While “Obese/Metabolic NAFLD” is associated with NAFLD and features of the MetS and an increased risk of type 2 diabetes and cardiovascular disease, NAFLD caused by I148M variant in PNPLA3 and the E167K variant in TM6SF2 is not accompanied by insulin resistance. Thus, lack of insulin resistance does not exclude NAFLD and not all patients with NAFLD are at increased risk of type 2 diabetes and cardiovascular disease. Given that both the MetS and the genetic variants in PNPLA3 and TM6SF2 are common, there are also many individuals with “double trouble NAFLD” [14].

### Future Research and Uncertainties

Although NAFL defined as macroscopic steatosis affecting >5% of hepatocytes predicts fibrosis [7,8,9], it is unknown how various degrees of steatosis predict liver outcomes. Such information would help the clinician to decide which patients to refer to the hepatologist. The same applies to the non-invasive markers of NAFL proposed to be used by the recent European NAFLD guideline if imaging tools are not available [101]. This guideline also recommended testing for the I148M gene variant in “selected cases and in clinical trials”. The latter might be helpful in identifying patients with NAFLD who are at risk for advanced liver disease but who lack features of the MetS and are therefore not at risk for cardiovascular disease or type 2 diabetes. A cost–benefit analysis of this suggestion is warranted.

## Figures and Tables

**Table 1:** Definitions of normal liver fat using different approaches.

Study	Year	*N*	Subjects	Normal Value
*Biochemical*				
Laurell S [21]	1971	3	Healthy subjects	2.0 g/100 g of dry tissue weight
Donhoffer H [15]	1974	107	Unselected cadavers	5.5 g/100 g of wet tissue weight
*Histology*				
Kleiner DE [17]	2005	576 + 162	Adults and children	Macroscopic fat in <5% of hepatocytes
Brunt EM [3]	2011	976	Adults	Macroscopic fat in <5% of hepatocytes
Bedossa P [19]	2012	679	Morbidly obese adults	Macroscopic fat in <5% of hepatocytes
*CT*				
Piekarski J [22]	1980	100	Healthy subjects	50–57 HU or 8–10 HU higher than spleen
*^1^H-MRS*				
Szczepaniak LS [23]	2005	345	Population-based, healthy subjects	<5.56%
Petersen KF [24]	2006	170	Healthy subjects	<3.0%
*MRI-PDFF*				
Fishbein MH [25]	1998	28	Healthy subjects	<9.0%
*US*				
Joseph AE [26]	1978	60	Adults referred to gastroenterologist	Absense of echogenicity or brightness of the liver
Saveymuttu SH [27]	1985	490	Adults referred to gastroenterologist	Absense of echogenicity or brightness of the liver

^1^H-MRS, proton magnetic resonance spectroscopy; CT, computed tomography; HU, Houndsfield Unit; MRI-PDFF, magnetic resonance imaging-determined proton density fat fraction; US, ultrasound.

**Table 2:** Insulin sensitivity in studies comparing liver fat between PNPLA3 I148M carriers and non-carriers.

Cohort	*N*	BMI (kg/m^2^)			Liver Fat			Insulin Sensitivity (HOMA-IR)			*S*-Triglycerides (mmol/L)		
		I148^II^	I148^IM^	I148^MM^	I148^II^	I148^IM^	I148^MM^	I148^II^	I148^IM^	I148^MM^	I148^II^	I148^IM^	I148^MM^
Multiethnic ^1^ [12]	2111	30.4	31.1	30.0	3.7% ^a^	4.6% ^a^	7.7% ***^,a^	3.3	3.5	3.3	1.32	1.35	1.41
		31.6	32.0	32.2	3.1%	4.8%	4.8% ***	3.3	3.3	4.4	0.97	0.97	1.02
		29.2	28.8	28.8	3.5%	3.7%	3.5% ***	2.3	2.4	2.0	1.25	1.21	0.90
Germany [79]	330	29.9	29.1	28.7	5.4% ^a^	6.0% ^a^	7.2% ***^,a^	12.6 ^v,z^	12.9 ^v,z^	12.9 ^v,z^	NA	NA	NA
Finnish [80]	291	30.5	30.0	32.2	9.0% ^a^	10.4% *^,a^	14.1% **^,a^	72 ^y,z^	70 ^y,z^	74 ^y,z^	1.82	1.60	1.52
British [81]	98	34.6	33.2	31.7	26.7% ^a^	28.8% ^a^	33.5% ^a^	2.4	3.1	2.6	1.60	1.70	1.40
Multiethnic ^2^ [82]	1214	NA ^×^	NA ^×^	NA ^×^	57 ^b^	55 ^b^	46 ***^,b^	NA ^×^	NA^×^	NA^×^	NA ^×^	NA ^×^	NA ^×^
					55	51	47 ***						
Dutch [83]	470	37.7	37.6	37.6	66% ^c^	78% ^c^	100% ***^,c^	2.7	2.8	2.9	1.42	1.47	1.46
Italian [84]	61	25.7	25.9		16% ^d^	32% *^,d^		3.4	4.7		1.13	1.15	
Italian [85]	253	30.7	30.7	29.8	44% ^c^	48% ^c^	63% **^,c^	3.9	4	5.2	1.64	1.85	1.79
Italian [86]	211	32.1	30.4	31.7	4 ^e^	4 ^e^	4 ^e^	3.5	3.5	2.8	1.77	1.59	1.26 **
Taiwanese [87]	879	23.3	23.6	23.6	13% ^f^	19% ^f^	23% *^,f^	1.4	1.5	1.5	1.11	1.16	1.38 *
South Korean [88]	1363	24.7	24.4	23.9 **	38% ^f^	45% ^f^	54% *^,f^	2.3	2.1	1.6 **	1.54	1.38	1.31 **
Taiwanese, pediatric [89]	520	26.3	26.2	25.9	21% ^f^	13% ^f^	30% **^,f^	2.4	2.5	1.7	1.11	1.03	0.94
Italian, pediatric [90]	475	NA	NA	NA	13% ^f^	19% ^f^	41% *^,f^	3.3	3.0	3.0	0.56	0.56	0.53
Italian, pediatric [91]	149	95.2 °	95.0 °	94.1 °	70% ^g^	7% ^g^	4% ***^,g^	2.5	2.7	2.4	1.28	1.19	1.39
					30%	78%	4%						
					0%	15%	92%						

BMI, body mass index; CT, computed tomography; HOMA-IR, Homeostasis model assessment of insulin resistance [62]; HU, Houndsfield Unit; MRI, magnetic resonance imaging; NA, not available; OGTT, oral glucose tolerance test; US, ultrasound. * Significant difference between groups in ANOVA or *t* test. * *p* < 0.05; ** *p* < 0.01, *** *p* < 0.0001. Data are presented as mean or median. ^1^ Caucasian, African and Hispanic Americans; ^2^ Hispanic and African Americas. ° BMI centiles; ^a^ ^1^H-MRS (liver fat content,%); ^b^ CT (liver density, HU); ^c^ Histology (prevalence of steatosis, %); ^d^ Histology (% hepatocytes steatotic); ^e^ US (severity of steatosis by Hamaguchi score, 3–4 = moderate); ^f^ US (prevalence of steatosis, %); ^g^ Histology (severity of steatosis, grade 1/2/3); ^v^ OGTT (arbitrary unit); ^y^ fasting serum insulin (pmol/L); ^z^ hyperinsulinemic clamp was also performed, data not shown in the table; ^×^ Data not shown, but it was reported that genetic variation at rs738409 did not correlate with HOMA-IR, insulin sensitivity index, BMI or *S*-triglycerides.

**Table 3:** Insulin sensitivity in studies comparing liver fat between TM6SF2 E167K carriers and non-carriers.

Cohort	*N*	BMI (kg/m^2^)		Liver Fat		Insulin Sensitivity (HOMA-IR)		*S*-Triglycerides (mmol/L)	
		EE	EK + KK	EE	EK + KK	EE	EK + KK	EE	EK + KK
Multiethnic ^1^ [92]	4587	29.6	28.5/31.8	3.5% ^a^	4.4%/15.7% ***^,a^	3.0	2.9/4.6	1.39	1.33/1.47 *
Finns [97]	300	33.7	32.5	6.8% ^a^	11.2% *^,a^	3.0	2.9	1.40	1.50
British [81]	98	32.6	35.4	28.5% ^a^	29.0% ^a^	2.7	4.0	1.60	1.50 *
Argentineans [13]	361	29.8	30.2	NA	NA	3.1	3.0	1.87	1.31
Multiethnic ^2^ [98]	502	32.2	31.2/30.8	S0: 3% ^b^	S0: 0%/0% ^b^	3.5	2.8/2.8	1.70	1.36/1.08 **
				S1: 50%	S1: 35%/45%				
				S2: 27%	S2: 40%/20%				
				S3: 20%	S3: 25%/35% *				
Multiethnic ^1^, pediatric [99]	957 ^	33.0	32.6	6.7% ^c^ ^	11.1% **^,c,^^	1.9 ^x^	2.0 ^x^	1.20	1.21
Italian, pediatric [100]	1010	2.9 °	2.9 °	47% ^d^	89% **^,d^	5.6	4.6	1.12	1.02 *

BMI, body mass index; BMI-SDS, body mass index standard deviation score; HOMA-IR, Homeostasis model assessment of insulin resistance [62]; MRI-PDFF, magnetic resonance imaging-measured proton density fat fraction; NA, not available; OGTT, oral glucose tolerance test; US, ultrasound; WBISI, whole body insulin sensitivity index. Significant difference between groups in ANOVA or *t* test, * *p* < 0.05; ** *p* < 0.01; *** *p* < 0.0001. Data are presented as mean or median. ^1^ Caucasian, African and Hispanic Americans; ^2^ Caucasian, Asian, Hispanic; International Liver Disease Genetics Consortium; ^ Liver fat content available on 454 subjects, BMI, insulin sensitivity and *S*-triglycerides on 957 subjects; ° BMI-SDS; ^a^ ^1^H-MRS (liver fat content, %); ^b^ Histology, prevalence of each steatosis grade; ^c^ MRI-PDFF, liver fat, %, (*n* = 454); ^d^ US (prevalence of steatosis, %); ^x^ OGTT (WBISI).

## Abbreviations

^1^H-MRS	proton magnetic resonance spectroscopy
ALT	alanine aminotransferase
AST	aspartate aminotransferase
BMI	body mass index
BMI-SDS	body mass index standard deviation score
CHD	coronary heart disease
CoA	coenzyme A
CT	computed tomography
DM	diabetes mellitus
DHS	Dallas Heart Study
FFA	free fatty acids
fS	fasting serum
HDL	high density lipoprotein
MCP-1	monocyte chemoattractant protein-1
HCC	hepatocellular carcinoma
HDL	high density lipoprotein
HOMA-IR	homeostasis model assessment for insulin resistance
LDL	low density lipoprotein
MetS	metabolic syndrome
MRI	magnetic resonance imaging
NAFL	non-alcoholic fatty liver
NAFLD	non-alcoholic fatty liver disease
NASH	non-alcoholic steatohepatitis
OGTT	oral glucose tolerance test
P	plasma
PDFF	proton density fat fraction
PNPLA3	patatin-like phospholipase domain-containing 3
TM6SF2	transmembrane 6 superfamily member 2
TNF-α	tumor necrosis factor-α
US	ultrasound
VLDL	very low density lipoprotein
